# Click Chemistry Methodology: The Novel Paintbrush of Drug Design

**DOI:** 10.1021/acschembio.4c00608

**Published:** 2024-12-27

**Authors:** Ioana Oprea, Terry K. Smith

**Affiliations:** Biomedical Science Research Complex, Schools of Biology and Chemistry, University of Saint Andrews, North Haugh, St Andrews KY16 9ST, United Kingdom of Great Britain and Northern Ireland

## Abstract

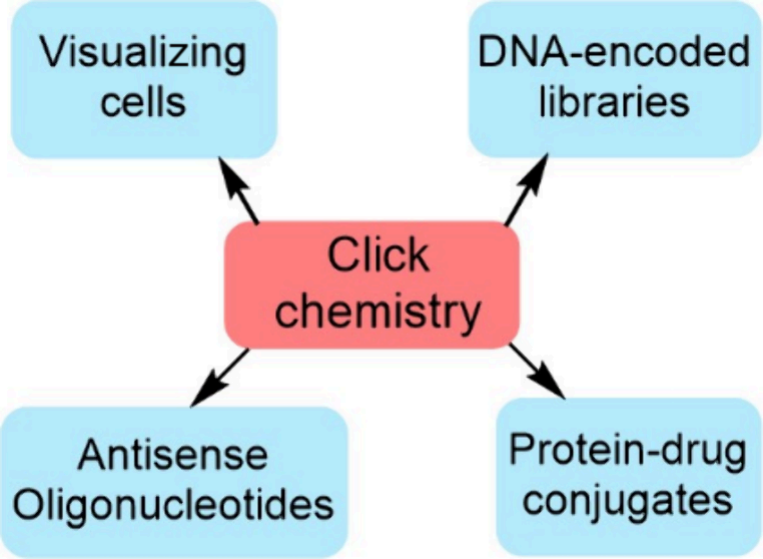

Click chemistry is an immensely powerful technique for the synthesis of reliable and efficient covalent linkages. When undertaken in living cells, the concept is thereby coined bioorthogonal chemistry. Used in conjunction with the photo-cross-linking methodology, it serves as a sound strategy in the exploration of biological processes and beyond. Its broad scope has led to widespread use in many disciplines; however, this Review focuses on the use of click and bioorthogonal chemistry within medicinal chemistry, specifically with regards to drug development applications, namely, the use of DNA-encoded libraries as a novel technique for lead compound discovery, as well as the synthesis of antisense oligonucleotides and protein–drug conjugates. This Review aims to provide a critical perspective and a future outlook of this methodology, such as potential widespread use in cancer therapy and personalized medicine.

## Introduction

**Figure 1:**
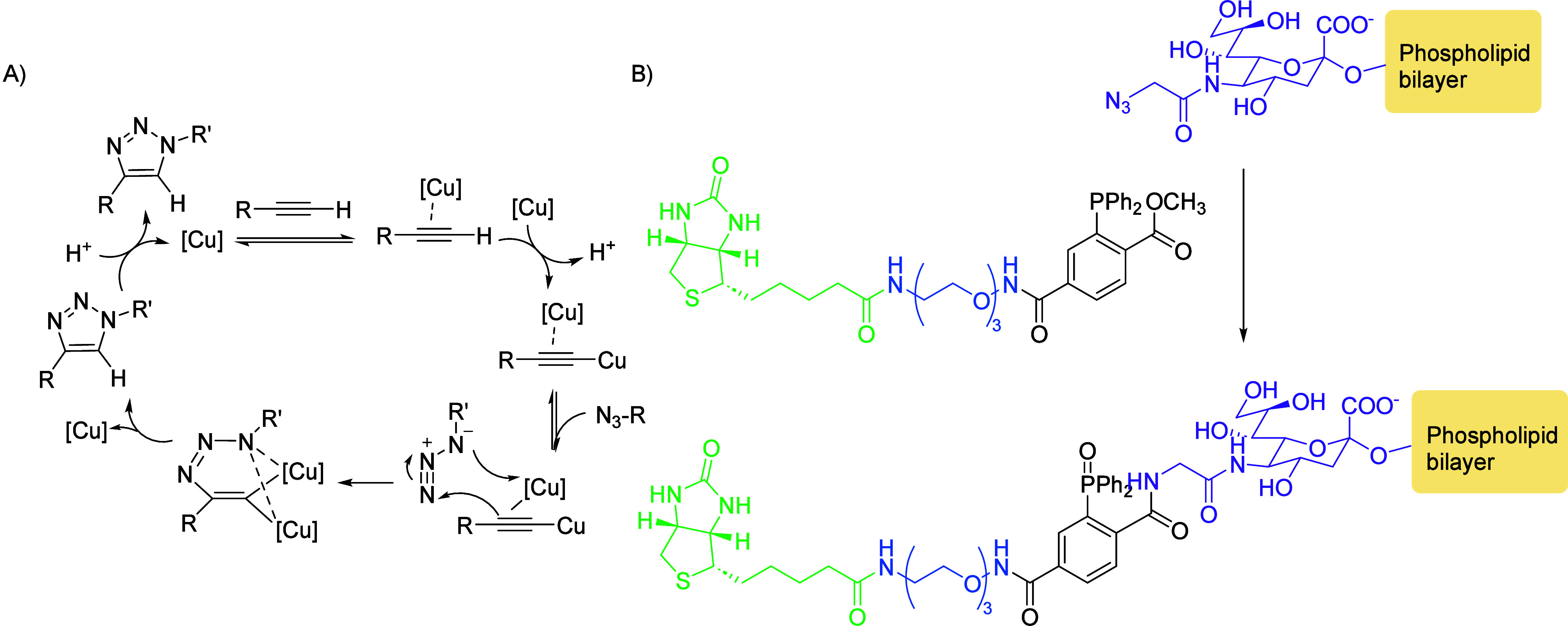
(A) The mechanism of CuAAC, adapted from WorellB. T. et al., Science2013, 340, 457–46023558174 10.1126/science.1229506PMC3651910. (B) Reaction of biotinylated (green) triarylphosphine (black) with azide containing-sialic acid residues (purple) on the cell surface via Staudinger ligation. Figure adapted from ref (3).

The Nobel Prize-winning concept of click chemistry, first proposed by Sharpless et al.,^1^ describes a class of stereospecific reactions with a high thermodynamic driving force under benign conditions that employ readily available reagents and generate stable products in a physiological environment, which are easily isolated and purified by affinity-chromatographic methods. This pioneering approach of considering highly reactive reaction components with narrow chemoselectivity profiles such that side reactions are avoided, that is, the reagents “click” together akin to a belt fastening mechanism, led to the discovery of a new combinatorial style of chemistry that serves to achieve sequential transformations of a broad scope. The copper(I) catalyzed Huisgen [2 + 3] azide–alkyne dipolar cycloaddition has become the representative click chemistry reaction following its independent discovery by Meldal et al.^2^ (Figure 1A); mechanistically, the copper catalyst inserts into the terminal alkyne to form a copper–acetylide complex, which is then followed by the addition to the azide. Despite its robustness as a click reaction, the toxicity of copper prevents its practical use in living organisms. Nevertheless, a biologically sound alternative was developed by Bertozzi et al.,^3^ namely a modified Staudinger reaction employing an azide and a triarylphosphine, the latter tethered to a neighboring electrophilic trap to form a stable amide bond via intramolecular cyclization with the aza-ylide intermediate, thereby circumventing its hydrolysis. This reaction was undertaken on sialic acid residues of glycoproteins on the surface of living Jurkat cells, which were metabolically engineered to contain an azide moiety. The visualization of the formed adduct was performed due to the incorporation of a biotin residue onto the phosphine analogue, thus enabling cell analysis via flow cytometry after staining with fluorescein isothiocyanate–avidin (Figure 1B). The functional groups involved in this process are abiotic, have finely tuned “click” reactivity for each other, and simultaneously exhibit no interaction with the preexisting functionality in the cellular environment. Bertozzi et al. coined the term bioorthogonality to describe these considerations, thus allowing for the use of the click chemistry methodology in living cells as a powerful molecule-building tool. The world of applications of the click chemistry methodology is vast, e.g., in protein target identification,^4^ visualizing cells, labeling DNA,^5,6^ protein assembly,^7^ cellular signaling,^8^ lipid messengers,^9^ and targeting cancer cells.^10^

This Perspective addresses the pioneering tactics of bioorthogonal chemistry, comparing the historical research as well as recent advancements in click reaction development to address the shortcomings of the former. The bulk of the Review focuses on a selection of innovative applications of these that play a significant role in drug discovery and development. These, as well as future innovations, will continue to expand the scope of click chemistry likely far beyond what was envisioned at its conception.

## General Methodology

**Figure 2:**
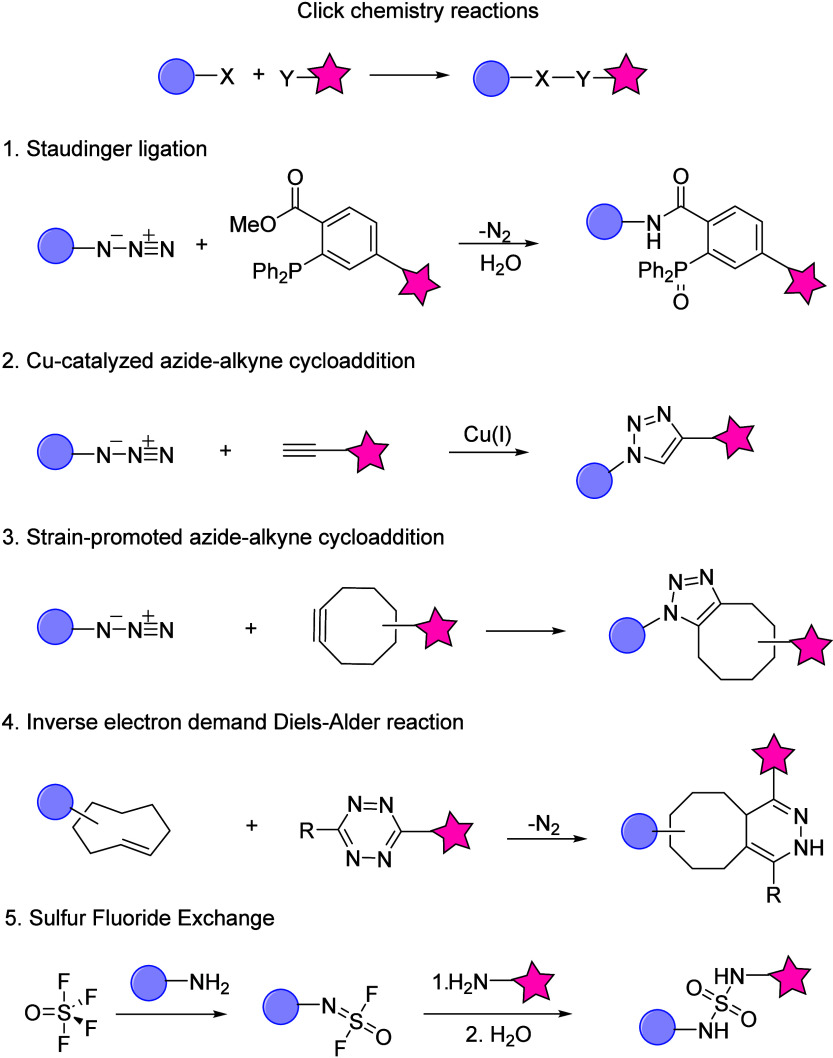
Click reactions: (1) Staudinger ligation, (2) CuAAC, (3) SPAAC, (4) IEDDA, and (5) SuFEx. Figure adapted from ref (16).

The methodology of employing click chemistry within a cellular environment follows a general outline, which involves the stepwise incorporation of a bioorthogonal group onto a protein of interest (POI) through biochemical means (e.g., expressed protein ligation,^11^ metabolic engineering,^12^ and tagging-via-substrate^13^) and its subsequent site-specific reaction with a small molecular probe^14^ via click chemistry (Figure 2).

### Click Reactions

The foremost consideration of this process is the choice of click reaction, of which four types are most widely used (Figure 2): the classic copper(I)-catalyzed Huisgen [2 + 3] azide–alkyne dipolar cycloaddition (CuAAC), copper-free azide–strained alkyne cycloaddition (SPAAC), inverse-electron demand Diels–Alder (IEDDA) reactions, and Staudinger ligation. While the original Staudinger ligation was the pioneering bioorthogonal click reaction, it posed several shortcomings, namely the nonspecific oxidation of phosphine reagents in biological systems and slow reaction kinetics, which hindered its use in the tracking of faster biological processes.

For comparison, IEDDA shows the fastest reaction rates (e.g., 2.2 × 10^4^ M^–1^ s^–1^ in MeOH), followed by CuAAC (10–10^4^ M^–1^ s^–1^ in DMSO/water). Moderate reaction rates were registered for SPAAC (<1 M^–1^ s^–1^ in MeOH), which places the Staudinger ligation as the slowest of the click reactions presented here (<8 × 10^–3^ M^–1^ s^–1^ in DMF/water).^15^

To avoid this, higher concentrations of the phosphine reagent can be used; however, this increases the background signal in fluorescence imaging.^16^ Conversely, inspired by the reduction of azides with triarylphosphines in water and their further modification by the insertion of an electrophile (such as an ester moiety) to yield stable amide products without a phosphine, the thus-termed traceless Staudinger ligation^3^ has become the preferred alternative.

The most widely reported click chemistry reaction within the cell is the strain-promoted azide–alkyne cycloaddition (SPAAC), thus circumventing the toxicity of copper as a catalyst *in vivo*.^17^ The azide is a popular and functional bioorthogonal handle, as it is kinetically stable, bioinert, and abiotic, and its insertion within a molecular target does not cause structural alterations due to its small size. Moreover, the strained cyclooctyne system can be further modified to improve reaction kinetics with respect to CuAAC by introducing electron-withdrawing abiotic substituents such as fluorine. An example of this approach used difluorinated cyclooctyne, which dramatically improved reactivity comparable to CuAAC for *in vivo* imaging of azide-labeled sugars.^18^ Further improvements to the hydrophilicity of the cyclooctyne component were also achieved by considering an innovative azacyclooctyne structure, 6,7-dimethoxyazacyclooct-4-yne, synthesized by Bertozzi et al. from glucose. Thus, due to improved bioavailability, nonspecific binding was minimized, resulting in an enhanced sensitivity of azide detection.^19^ It should be noted that there are restrictions in the use of SPAAC, primarily due to the risks of the cyclooctyne reagents reacting with cellular and plasma nucleophiles such as thiols, thus limiting the type of targets.^20^

The inverse electron-demand Diels–Alder (IEDDA) reaction of electron-withdrawing dienes (typically tetrazines) with electron-donating dienophiles (e.g., alkenes and alkynes) provides a different alternative to SPAAC, outperforming it with regards to reaction kinetics (driven by ring distortion release and nitrogen production), better chemoselectivity, and exceptional biocompatibility.^21^ The pioneering IEDDA reaction between 1,2,4,5-tetrazines (Tz) and *trans*-cyclooctenes (TCO) has been further improved so far as to reach kinetics 10 000-fold those of CuAAC (with second order rate constants up to 3.3 × 10^6^ M^–1^ s^–1^),^22^ enabling ligation at very low concentrations and overcoming issues such as rapid excretion *in vivo*;^23^ therefore, it is increasingly harnessed in nuclear medicine, with notable applications such as tumor pretargeting^24^ and biocompatible polymer synthesis.^25^ Notably, IEDDA also facilitates the investigation of unsaturated free fatty acids in living cells, as minimal structural alterations to the biochemical properties of said lipids would be introduced. By modifying the reagent choice, such as utilizing Tz and cyclopropenes instead of TCOs, fast ligation kinetics were achieved, enabling visualization using live-cell microscopy (Tz-fluorophore) and proteomic analysis.^26^

Recent advancements in the development of click reactions for fine-tuned bioorthogonality and improved probe design span a variety of innovative reactions, of which sulfur–fluoride exchange (SuFEx), developed by Sharpless et al.,^27^ is employed with increased frequency (Figure 2). SuFEx involves sulfur(VI) fluorides, e.g., SOF_4_-derived iminosulfur oxidifluorides, which are stable *in cellulo* but react with a range of nucleophiles to form S–N, S–O, and S–C linkages in a click-like fashion, affording combinatorial SuFEx libraries. Specifically, examples of biocompatible SuFEx chemistry in dilute aqueous solutions and under mild conditions of temperature and pH (25 °C, pH 7.3) were achieved. This powerful technique has in recent years been used for drug design,^28^ activity-based profiling,^29^ and protein-targeting studies,^30^ to name a few remarkable applications; moreover, these novel advancements appoint SuFEx reactions as a favorable contender for DNA-encoded library (DEL) technology, which will be presented in more detail in the eponymous section.

### Photo-Cross-Linking and Choice of Probes

**Figure 3:**
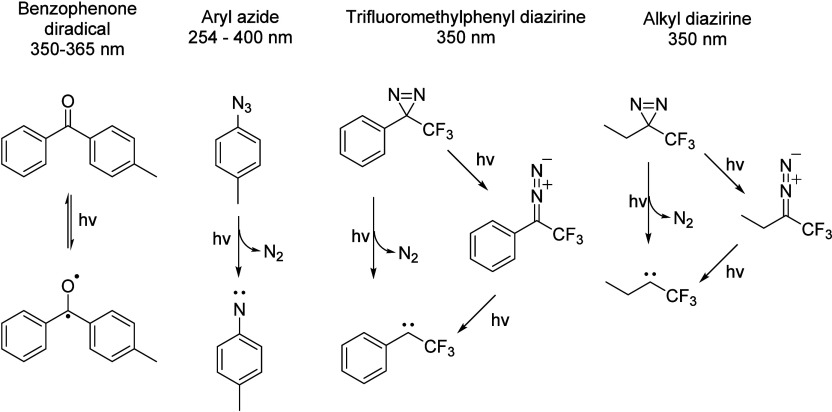
Mode of action of different photo-cross-linkers: benzophenone, aryl azide, trifluoromethylphenyl diazirine, and alkyl diazirine. Figure adapted from ref (34).

This procedure also generally employs *in situ* photoaffinity labeling (PAL) to visualize and monitor specific proteins of interest (POIs).^31^ PAL implies the formation of a new covalent bond (“cross-link”) to proximal amino acid residues on the POI as a result of photoirradiation of a photo-cross-linking functional group (e.g., benzophenone,^32^ aryl azide, and diazirine,^33^Figure 3), generating a highly reactive species that interacts spatioselectively with the probe, which is a modified analogue of a natural binding partner (target specific ligand) to the POI. The analogue is thus engineered to contain a functional group able to perform click chemistry, as well as a reporter group, which is a nonactive moiety that enables visualization of the product, e.g., biotin or a fluorophore.^34^

The general criteria required for a photoaffinity probe (PAP), aside from the ability to generate highly reactive intermediates upon irradiation, lists several considerations: structural resemblance to the pharmacophore target with similar affinity, leading to the formation of a stable adduct, and critically a selective wavelength activation that does not cause damage to the biological system.^35^ Among the most common photoreactive groups, benzophenone-based PAPs require activation with light at 350–360 nm, forming a reactive carbonyl triplet state that reacts with POIs via a sequential abstraction–recombination mechanism, with high affinity toward methionine.^34,36^ Despite limited protein degradation, the steric bulk of the phenyl groups could impact binding to the respective targets, and the risks of nonspecific labeling during the long irradiation times required may disfavor the use of benzophenone as a PAP.^37^ Similarly, aryl azides (phenyl- and nitro-substituted) are activated by short-wavelength UV (under 300 nm), forming nitrenes that interact nonspecifically with target proteins^38^ due to the risk of rearrangement into azacycloheptatetraene,^39^ inducing nonspecific labeling following reactions with other nucleophiles. Instead, diazirine-based PAPs (notably trifluoromethyl phenyl diazirine) may constitute a more favorable alternative, as they are inert toward nucleophilic attack and have excitation wavelengths within 350–380 nm, which is not harmful; upon irradiation, they generate reactive carbenes,^40^ which achieve more specific labeling due to their short lifetime, as only the desired target POI lies within the required proximity for bonding.^41^ Aliphatic diazirines do, however, have the caveat of being less stable than their aromatic analogues, thereby causing nonspecific photolabeling.^42^ Among all three classes of photo-cross-linkers, benzophenone has the distinct property of allowing repeated photoactivation due to the formation of a biradical.

**Figure 4:**
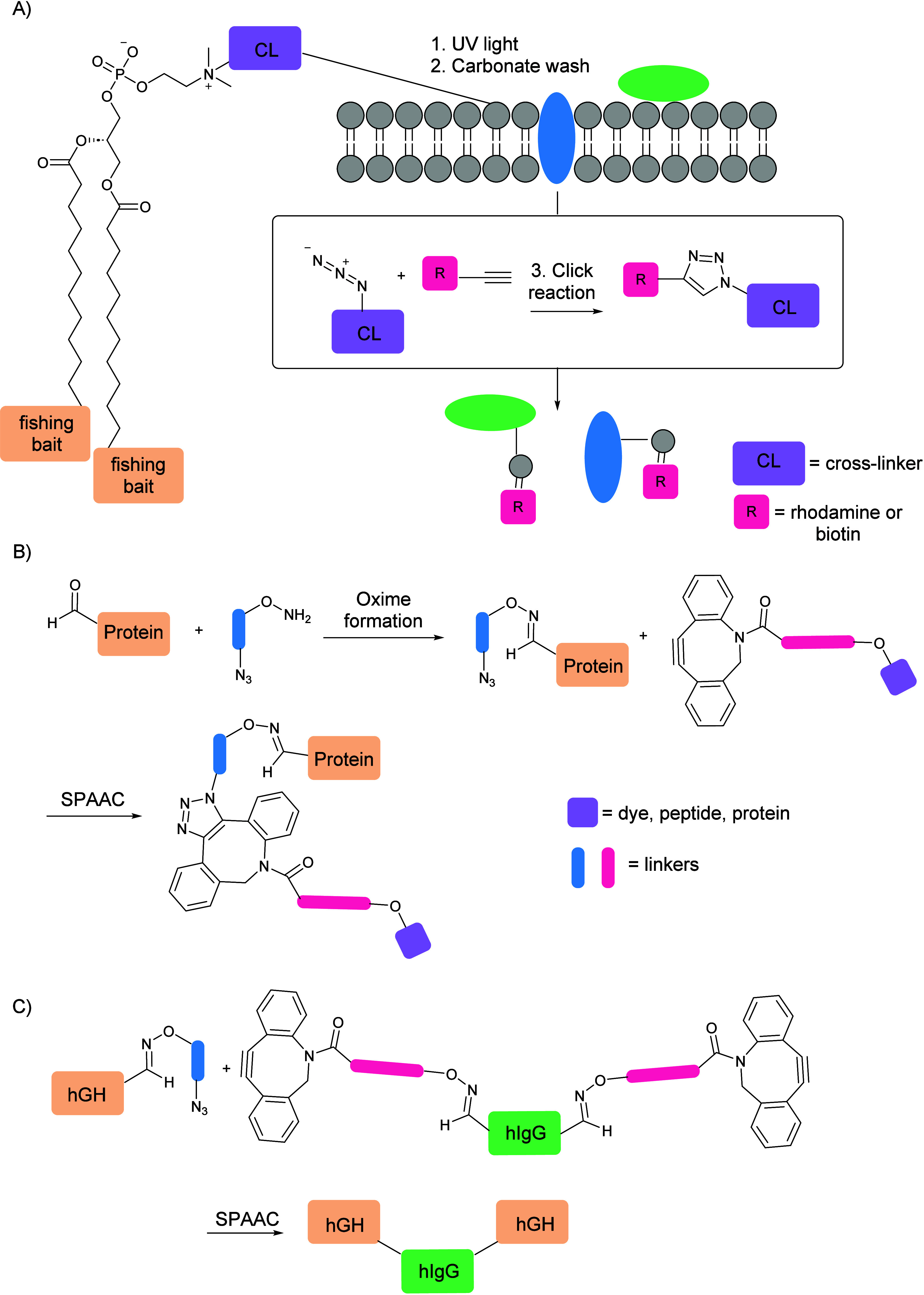
(A) Engineered phosphatidylcholine analogues containing an azide moiety at the hydrophobic end, first photo-cross-linked to proteins interacting with the phospholipid bilayer due to photoaffinity labels present on the hydrophilic end. The adduct formed further underwent CuAAC with a rhodamine–alkyne conjugate, allowing for fluorescence scanning. (B) Site-specific protein functionalization was achieved by the initial modification of the aldehyde moiety to a stable oxime via a reaction with an aminooxy reagent containing an azide group, which further reacted via SPAAC with a dibenzoazacyclooctyne fluorophore conjugated onto a different protein. (C) Synthesis of heterobifunctional protein conjugates achieved by employing the methodology illustrated in (B) utilizing hGH- and hIgG-functionalized proteins. Figure 4. (A) Adapted from ref (43). Figures 4. (B, C) Adapted from ref (44).

To showcase the versatility of this approach in chemical proteomics studies, we will explore a few examples. For instance, Grubbens et al. employed this methodology for the specific detection of proteins interacting with the phospholipid bilayer by photo-cross-linking these *in situ* to engineered lipid analogues mimicking membrane phospholipid phosphatidylcholine within inner mitochondrial membrane vesicles of *Saccharomyces cerevisiae*.^43^ The analogues were designed to contain a photoaffinity label (phenylazide or benzophenone) at the hydrophilic head of the phospholipid and an azide functionality at the hydrophobic end, which underwent click chemistry with a tetramethylrhodamine–alkyne conjugate, allowing for fluorescence scanning of the adduct and subsequent identification of separated POIs by mass spectrometry (Figure 4A). In addition to to applications in proteome analysis, this methodology was also employed in state-of-the-art protein assembly developed by Bertozzi et al.^44^ Site-specific protein functionalization was achieved using the formylglycine-generating enzyme (FGE), which oxidizes the sulfhydryl group within cysteine residues of formylglycine (fGly) during protein expression in *E. coli* or mammalian cells. The resulting aldehyde functionality was subsequently modified via reaction with an aminooxy reagent to give a stable oxime. The aminooxy reagent used further served as a small-molecule linker, as it was chosen to contain an azide moiety; this functional group further reacted via SPAAC with a dibenzoazacyclooctyne fluorophore conjugated onto a different protein. The specific synthesis of heterobifunctional protein conjugates of significant complexity was thereby achieved, e.g., a full-length human IgG coupled to either human growth hormone (hGH) or to maltose-binding protein; the former potentially improves the serum half-life of protein therapeutics, while the former enables dual binding of a single molecule (Figure 4B, C).

Novel fluorophores, also coined click-activated luminogenic fluorophores (CalFluors), have since been developed by Bertozzi et al.^45^ These are universal switches capable of being internally quenched via photoinduced electron transfer in the azide form but unquenched upon generation of the triazole. The core motif is a 3-azido-4,6-dialkoxyaryl group, which when incorporated into xanthenes (and functionalized with zwitterionic solubilizing groups to obviate washing steps) generates a plethora of dyes emitting from green to far red wavelengths, which are widely used in a variety of *in vivo* applications.

## Applications

### DNA-Encoded Libraries

**Figure 5:**
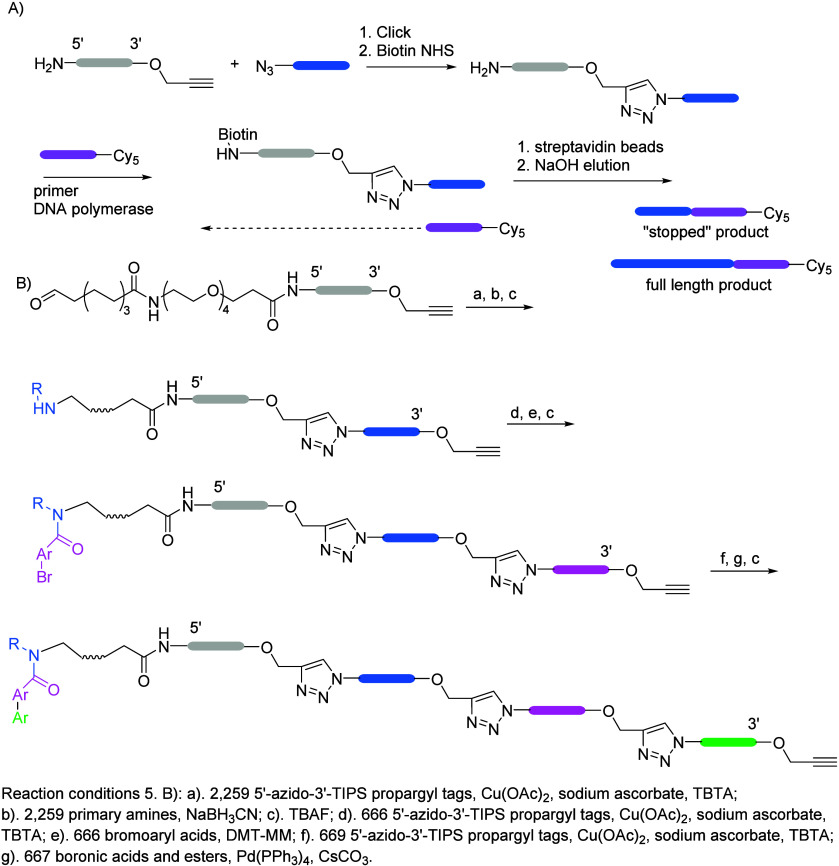
(A) Scheme for substrate synthesis and read-through study; following CuAAC, the oligonucleotide formed was labeled with biotin at the 5′-end and a complementary primer to the 3′-terminal region (Cy-5-labeled 17-mer primer) that underwent extension by DNA Polymerase I. (B) Scheme of a chemical ligation-based library synthesis strategy; templates with multiple triazole junctions were synthesized following the reaction conditions outlined above.

Lead compound discovery poses a significant challenge in the field of drug development. Click chemistry, due to its impressive specificity, has had a significant impact on this process by advancing the screening of compound libraries as a result of the ability to do so directly from within the compound mixture through high-throughput methodology.^46^ Recent advancements in technological development of this kind have shifted toward the use of DNA-encoded libraries (DELs, Figure 5). These are combinatorial libraries of molecules that are of promisingly similar structure to drug molecules, each with an attached “barcode” comprising a DNA sequence that encodes the compound’s structure.^47^ The advantage of this method is foremost the ever impressive diversity and the efficiency of screening for ligands of a specific protein target. Initial combinatorial biochemical methods such as phage display^48^ or RNA display^49^ emerged as discovery methods for biopolymer ligands to biological targets by using an environmental selection method analogous to that of biological evolution: select species carry oligonucleotide “gene” encoding the structure, which is thus used for its amplification; sequencing the gene allows for species identification.^50^ This has amassed significant interest toward its potential broader scope within synthetic small-molecule drug leads, which would increase current screening throughput within the pharmaceutical industry by orders of magnitude; for scale, libraries with over 800 million-member DNA-encoded small molecules have thus been successfully achieved.^50^ DNA-based multistep synthesis has been reported^51^ via the formation of covalent linkages between the encoding DNA and the small-molecule building blocks (positioned appropriately through Watson–Crick base pairing for single-stranded DNA templates^52^ or through Hoogsteen base pairing within the major and minor grooves to bind reactants, for double stranded DNA templates, that are less likely to interfere with the binding to enzyme targets^53^). The limitations of reaction compatibility with DEL technology (high yielding reactions, aqueous conditions, high chemoselectivity) essentially align with click chemistry requirements; in fact, CuAAC was first employed by Chen et al.^54^ in this context, but further advancements have used CuAAC for encoding tag ligation instead of enzymatic methods,^55^ as chemical ligation offers more flexibility toward sequence design. The stepwise example procedure (as a read-through study) is shown in Figure 5A: following CuAAC, the oligonucleotide formed was labeled with biotin at the 5′ end, and a complementary primer to the 3′-terminal region (Cy-5 labeled 17-mer primer) that underwent extension by DNA polymerase I (the Klenow fragment was the most efficient) was therefore attached. The removal of the triazole-linked template strand further simplified analysis of the products by LCMS.^55^ Iterative ligation afforded alternating azide and alkyne tags while protecting the alkyne with the TIPS group; the tags thus followed the motif 5′-azido-TXXXXXXXXXXXXXU-3′-propargyl-TIPS. Therefore, templates with multiple triazole junctions were achieved and further used in library synthesis, shown in Figure 5B: after chemical ligation with CuAAC, reductive amination, removal of TIPS by TBAF, CuAAC tagging, and acylation with bromoarylcarboxylates, Suzuki cross-coupling with boronate ester oligonucleotides (extra tags were used to encode products of reaction failure) and purification by reverse-phase HPLC yielded a 334 million compound library, which was subjected to affinity-mediated selection against the target soluble epoxide hydrolase (sEH), whose inhibitors could have potential interest in the treatment of COPD, cardiovascular disease, and even diabetes.^56,57^ Generally, measuring the fidelity of the reaction in DEL synthesis has been assessed using qPCR (to quantify DNA post synthesis and detect DNA damage), HPLC (synthetic yield), and gel electrophoresis (ligation efficiency).^58^

One downside to this approach is that side reactions with DNA tags pose the risk of compromising the library selection analysis during sequencing, as DNA damage could easily be achieved due to its reactive components (e.g., reactive heteroatoms on nucleobases, nucleophilic 3′-OH, glycosidic linkage, phosphodiester backbone) as well as harsh reaction conditions (e.g., high temperature, low pH) which can lead to depurination and strand scission.^59^ One way to avoid these consequences is careful consideration of reaction step sequences in order to limit protection strategies or change these (e.g., using TEA over TBAF in the deprotection of silyl ethers)^60^ to minimize the impact on DNA amplifiability; using the click Staudinger ligation has been shown to proceed with high yield and negligible degradation after overnight incubation.^61^

**Figure 6:**
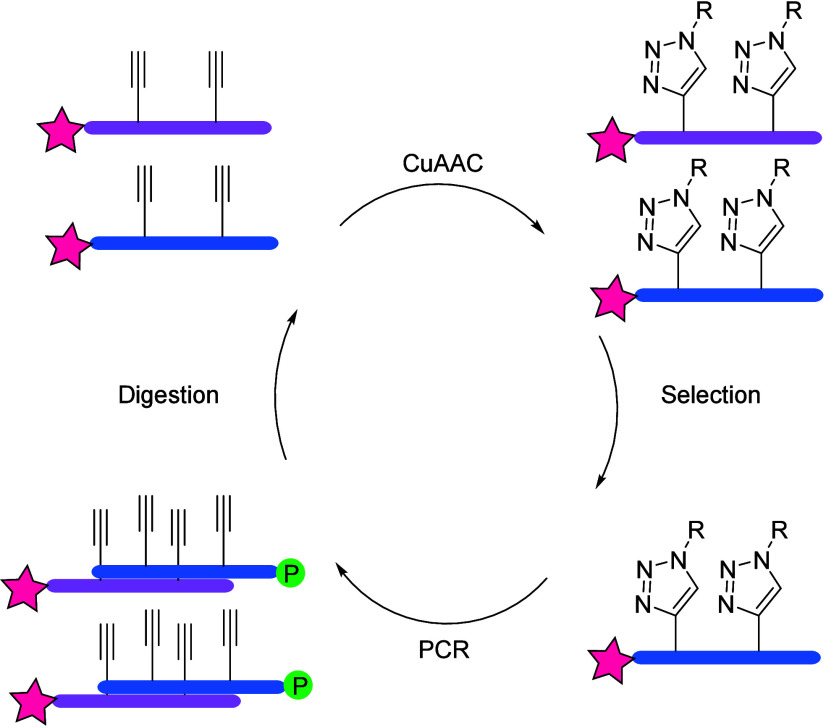
Click-SELEX process: alkyne-modified DEL starts the cycle, followed by CuAAC, incubation with fluorescent C3-GFP, wash, elution, and PCR amplification with EdU. Final regeneration of the library was achieved via digestion with λ-exonucleases and azide incorporation. Figure adapted from ref (62).

Another approach to utilizing click chemistry as a tool in DEL synthesis is the click-SELEX (systematic evolution of ligands by exponential enrichment) process, which is an adapted SELEX process by Mayer et al., as a combinatorial technique to produce oligonucleotides (commonly referred to as aptamers) that specifically bind to a target ligand.^62^ In particular, the synthesis of an alkyne-modified DEL was prepared by Mayer et al. through the replacement of thymidine nucleobases with C5-ethynyl-2′-deoxyuridine (EdU) followed by CuAAC functionalization with an azide component (chosen as 3-(2-azidoethyl)indole), yielding a modified DNA library. This was subsequently incubated with the target (chosen as cycle three green fluorescent protein (C3-GFP), which allowed for direct visualization); the remaining unbound molecules were washed away prior to the elution of the products with imidazole. The resulting molecules were amplified by PCR with EdU, and the single-stranded alkyne modified DNA was once again regenerated via digestion of the products with λ-exonucleases (hydrolyzing the 5′-phosphorylated strand of the double stranded DNA). The final step thus rebuilt the starting DEL by reaction with the azide moiety, allowing for the continuation of the cycle (Figure 6). This method avoids enzymatic incompatibility due to size restrictions of larger nucleobase replacements. Following 15 completed selection cycles, cloning, and sequencing of the DNA library, analysis was performed on the resulting dominant sequence family using flow cytometry (using 5′-Cy5 fluorophore); remarkable specificity to the target was observed, and SAR studies showed the dependency of binding on the indole moiety. As this was introduced via CuAAC, Mayer et al. proposed the term “clickmers”, thereby highlighting the importance of click chemistry in the success of this approach.

Further improvements to the outlined procedure were conducted, as Seela et al. showed the possibility of partial oxidation of the ethynyl-moiety under alkaline conditions to yield the corresponding ketone, which is incompatible with subsequent click functionalization and thus may lead to inhomogeneity.^63^ As a result, Mayer et al. modified the procedure to solid-phase synthesis, with DNA undergoing CuAAC while still attached to the solid support, followed by deprotection and purification; a full conversion to the desired product was thereby achieved, thus describing a larger-scale production protocol of nucleobase-modified nucleic acids.^64^

Despite the orthogonality and high-yielding nature of CuAAC, Cu(I) has been shown to introduce oxidative damage to DNA,^65^ which can be avoided by optimizing the ligands and reducing agents employed. Specifically, utilizing an *in situ* reducing agent such as sodium ascorbate and chelating ligands such as [tris(3-hydroxypropyltriazolylmehtyl)amine (THPTA) to improve the water solubility and to capture reactive oxygen species has been shown to improve CuAAC conditions.^66^ Even further optimizations to the ligand sphere, such as using a tris(triazolylmethyl)amine ligand, e.g., BTTAA and BTTES, promote the acceleration of the cycloaddition in living systems and improve the solubility of the catalyst complex at physiological pH.^67^

The issue remains that the availability and diversity of azides and alkynes starting blocks are not as prevalent in comparison to amines and alcohols, for example; therefore, biocompatible SuFEx technology may prove to be of interest in labeling DNA. An example of this endeavor was accomplished by Sharpless et al.,^68^ who proved the efficiency of a SuFEx reaction linking small molecules to amine-tagged single-stranded DNA featuring a long chain primary amine tail overhang and hairpin structure. Though few such instances have been reported, the applicability of this approach is clear and may be increasingly used in DEL synthesis.

Conversely, the PAC-fragment approach, coined by G. Liu,^69^ describes a novel methodology featuring photoaffinity labeling for screening small compounds or fragments against a target protein. Contrasting with high-throughput screening of larger compounds, low molecular weight structures are not sterically prevented from binding while carrying photoactivatable moieties such as diazirine, which is utilized to identify active sites. Essentially, photoactivated covalent capture of DNA-encoded fragments identifies hits binding to a target; each molecule in the library (synthesized using a split and pool fashion) includes a DNA encoding system, a linker of variable lengths containing the diazirine moiety, and a fragment potentially capable of covalent binding to the target. PCR amplification and DNA sequencing facilitate the identification of the fragments that successfully bound to the target, similar to classic DEL methodology.

### Antisense Oligonucleotides by Click Chemistry

**Figure 7:**
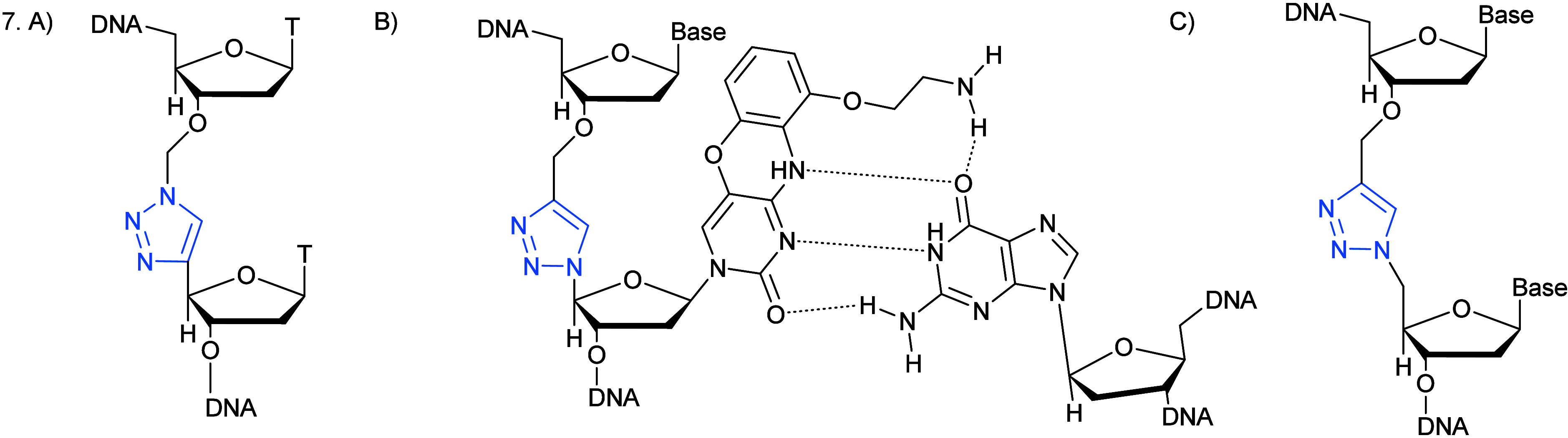
(A) Triazole-modified oligonucleotide successful at adopting the A-conformation required for RNA recognition. (B) Triazole G-clamp including an aminoethylphenoxazine nucleobase, thus modified to improve base pairing around the click modification base paired with guanine in complementary DNA. (C) Triazole-modified oligonucleotide, the first report of a synthetic DNA analogue with a modified backbone linkage shown to successfully undergo RNA transcription. (A, B) Adapted from BrownT. et al. Chem. Rev.2021, 121, 7122–715433443411 10.1021/acs.chemrev.0c00928. (C) Taken from ref (86).

Antisense oligonucleotides (ASOs) are synthetic short nucleic acids that can alter cellular RNA and therefore impact gene expression by affecting translation, thus targeting the source of the disease as opposed to downstream effects.^70^ Due to their sequence specificity, selective inhibition of genes can be achieved, minimizing toxic side effects and thereby making ASOs a promising avenue in the development of personalized drugs, some of which that target a number of genetic diseases have recently been approved.^71,72^ However, advancements to this field are still being undertaken, with significant improvements to therapeutic properties of ASOs spanning modifications to the sugar–phosphate backbone adopted through click insertion of triazoles, thereby increasing their stability toward degradation and improving cellular uptake by reducing anionic charge. Among the structures investigated, a 1,4-triazole (Figure 7A),^73,74^ was successful at adopting the A-conformation required for RNA recognition; further improvements to the base pairing around the click modification included the addition of an aminoethylphenoxazine nucleobase (termed G-clamp, promoting enhanced pairing with guanine, Figure 7B) at the 3′-position of the triazole-containing nucleobase.^75^ Introduction of a mismatch nearby strongly destabilized the DNA duplex, which would suggest activity as a potential mismatch sensor.^76^ Alternatively, modifications to the sugar moiety of the DNA backbone with ribose analogues^77^ or conformationally restricted locked nucleic acids (LNAs)^78^ yielded similar outcomes, advantageously doing so independently of the neighboring nucleobases. Conversely, enhanced stability and stronger binding of DNA–RNA duplexes was observed for the sugar modifications due to a stronger conformational lock; moreover, both the G-clamp and LNA modifications in combination with triazole insertion were successfully replicated by DNA polymerase, thus making this class of ASOs a potential solution to diseases involving issues in DNA amplification.

Introducing click-functional groups in nucleic acid scaffolds implies synthetic routes modifying either the phosphate group, the 5′-, 3′-, or 2′-OH of the ribose sugar, or the C5 position of pyrimidines. CuAAC is the preferred click reaction for this purpose, thereby conjugating the alkyne onto the oligonucleotides and utilizing azide-probes. Carell et al. developed a solid-phase synthesis utilizing the phosphoramidite derivatives (as more stable phosphate esters) of 2′-deoxiuridine-modified nucleosides 5-ethynyl-dU (dU^e^) and 5-(1,7-octadiynyl)-dU (d*U*°), which were prepared by Pd-assisted Sonogashira cross-coupling.^79^ The alkyne moiety was therefore introduced onto the DNA structure at the C5 position of each deoxyuridine, and the resulting 5′- dimethoxytrityl-nucleosides were reacted with 2-cyanoethyl *N*,*N*,*N*′,*N*′-tetraisopropylphosphorodiamidite; a number of azide click partners were chosen (fluorescein azide, an azido-sugar and a coumarin azide), illustrating efficient coupling.^80^ However, the dU^e^ group requires silyl protection during phosphoramidite nucleotide synthesis, making it a less favorable alternative to d*U*°; conversely, dU^e^ triphosphate is a superior substrate for DNA polymerases (similarly to dNTPs) in the context of amplification of long DNA templates.^81^ Further work on alkyne nucleobase derivatives was undertaken by Seela et. al, who reported an increase in the DNA helix stability following octadiynyl insertion with respect to an ethynyl modification; moreover, the group also investigated a synthetic derivative of the naturally occurring ribonucleoside inosine (7-alkynyl-7-deaza-2′-deoxyinosine) due to its ability to form wobble base pairs at ambiguous positions of tRNA anticodons (due to the lack of an amino group at the guanine 2-position), thus making it a “universal” nucleoside.^82^ 7-Deazapurines also showcased better stability at acidic pH, which promotes them as candidates for DNA footprinting.^83^ An alternative synthetic route to install the alkyne moiety onto nucleobases circumventing Sonogashira cross-coupling was developed by Hocek et al. by coupling a natural thymidine nucleoside to flexible alkynes (e.g., the hydrophilic propargyl-diethylene glycol (PEG) or hydrophobic undecyne (UN) linkers) via radical bromination as follows: treatment of the diacetoxy-protected thymidine with *N*-bromosuccinimide (NBS) and azobis(isobutyronitrile) (AIBN), followed by reaction with alcohol-derivatized PEG and UN linkers. The resulting nucleosides were then deprotected and converted into the triphosphate or phosphoramidite monomers for solid-phase synthesis toward oligonucleosides.^84^

Further modifications to the phosphate backbone were explored by Caruthers et al., in order to label specific positions therein, by generating ethynyl phosphinoamidites; the alkyne moiety was similarly functionalized via CuAAC with various azides to produce a substituted 1,2,3-triazolyl phosphonate-2′-deoxyribonucleotide internucleotide linkage, which was found to be highly resistant toward 5′- and 3′-exonucleases. This is advantageous for the main reason that base pairing and hybridization remain unaffected by the triazole insertion, as the nucleobases were not modified, allowing for a broader scope of this approach.^85^

The triazole–modified DNA backbone motif was analyzed by Brown et al. within the context of DNA-templated RNA synthesis to assess biocompatibility.^86^ Therefore, *in vitro* transcription of the modified oligonucleotide (Figure 7C) was successfully carried out using T7 RNA polymerase, an enzyme commonly used in biotechnology for the production of small RNAs. An *E. coli* growth inhibitor (54-mer DicF sequence) was transcribed using two templates, which were designed to include the modified triazole as a phosphodiester surrogate at the 4- or 5-position of the T7 promoter locus or within the coding sequence of the gene. The first template yielded no RNA product, which was due to a possible steric disruption of the DNA–protein complex; conversely, the second template yielded ∼80% of RNA compared to native control, which was further proven by mass spectroscopy. This first report of RNA transcription using a synthetic DNA analogue with a modified backbone linkage serves as a starting point for investigations within the RNA context and hopefully further into the production of “clicked” antisense oligonucleotides.

### Construction of Protein–Drug Conjugates Using Click Chemistry

**Figure 8:**
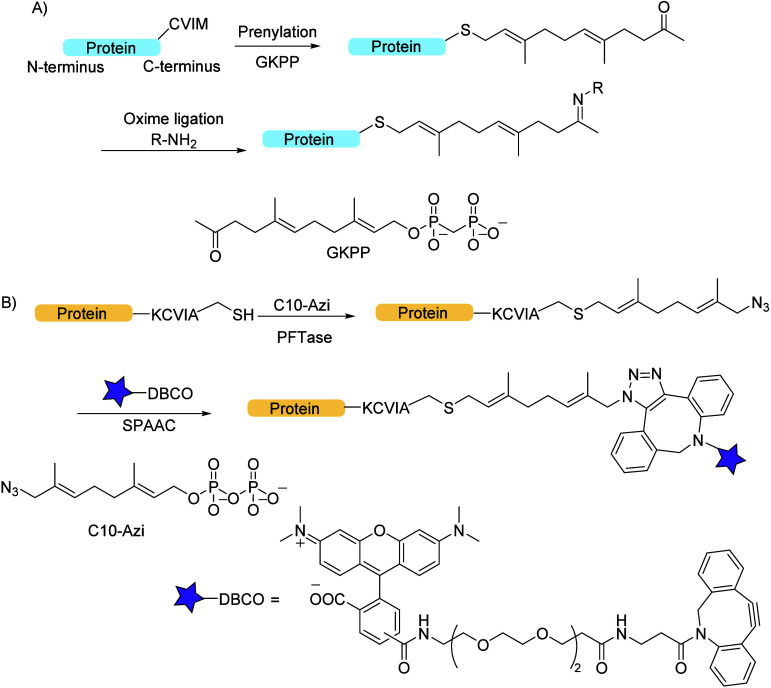
(A) *S*-Prenylation and click chemistry methodology employed in the synthesis of protein–drug conjugates: the repebody engineered to contain a C-terminal CVIM motif was *S*-prenylated using FTase, followed by oxime ligation with aminooxylated MMAF to yield a repebody derivative–drug conjugate. (B) *S*-Prenylation with the DARPins methodology: enzymatic labeling of DARPins engineered with a C-terminal CVIA sequence to a C10-Azi analogue by using PFTase and conjugation with DBCO-TAMRA using SPAAC. (A) Adapted from ref (104). (B) Adapted from ref (107).

Targeted therapies based on protein–drug conjugates have increasingly attracted attention as promising alternatives to classic anticancer drugs, as they involve the conjugation of cytotoxic drugs to protein binders, thereby delivering the active antitumor agent into specific cancer cells followed by drug release.^87^ This significantly improves the therapeutic window due to the increased potency and selectivity, as well as the delivery efficiency.^88^ The construction of protein–drug conjugates employs a vast array of proteins; their natural cellular modifications are exploited in order to enable enzymatic labeling and further site-specific drug conjugation (Figure 8).

Among these, post translational modifications (PTMs) of proteins occur as a means to enable or diversify their biological function, cellular localization, and activity;^89^ of these, protein–lipid modifications describe the attachment of hydrophobic molecules that carry out essential tasks (such as regulating protein trafficking and mediating protein–protein interactions).^90^ Cytoplasmatic PTMs of interest in this Review include *N*-myristoylation (which describes the attachment of the 14 carbon saturated fatty acid, myristic acid, on N-terminal amines of glycine residues, forming stable and irreversible amide bonds), as well as *S*-prenylation (involving the formation of a thioether bond between an isoprenoid molecule and the thiol group in cysteine residues in the vicinity of the C terminus of proteins).

In the former case, *N*-myristoyltransferases (NMTs) catalyze the binding of the activated myristoyl-CoA form,^91^ which causes a conformational change and subsequent protein binding.^92^ As there is no expression of NMTs present in prokaryotes, they utilize host mechanisms to perform *N*-myristoylation during infection, which some pathogenic eukaryotes also require, prompting interest toward pathogen-specific NMT inhibitors to attenuate viral infectivity in the treatment of disease.^93,94^ As a key regulator in protein stability, activity, and protein–protein interaction linked to immunity, autophagy, infection, and cancer,^90,93,95−97^*N*-myristoylation and its accompanying research in the context of drug targets constitute an important topic of interest. In fact, human and parasitic NMT-selective inhibitors pose significant interest as drug targets against cancer and even infectious diseases, such as malaria.^98,99^ In this context, Tate et al. were the first to develop a labeling method involving the incubation of azide-modified myristate analogues with a peptide mimicking the N-terminal myristoylation target motif Gly-XXX-Ser within the N-terminal region of the enzyme *Plasmodium falciparum* ADP ribosylation factor 1 (PfARF1) and a catalytic quantity of NMT cloned from *Candida albicans* (CaNMT).^100^ The enzymatic reaction yielded an azide-functionalized peptide, which further reacted via click Staudinger ligation with a capture reagent containing a phosphine moiety and a biotin label, allowing for visualization of the product following pull-down with avidin-beads. Transfer efficiency was quantified by immunoblot to be >99%, showing promise in proteomic applications and beyond.

*S*-Prenylation involves the attachment of an isoprenoid, either a short chain farnesyl (from the native farnesyldiphosphate, FPP, catalyzed by farnesyltransferase, FTase) or a longer chain geranylgeranyl group (from the native geranylgeranyldiphosphate, GGPP, catalyzed by geranylgeranyltransferase type I, GGTase-I) to cysteine residues.^101^ The FTase recognition sequence entails a terminal CaaX motif, where “C” is the cysteine residue that is selectively modified, “a” is aliphatic amino acids, and “X” is an amino acid which determines whether the protein is a substrate for FTase (X = Ala, Ser, or Met) or GGTase-I (X = Leu, Ile, or Phe).^102^ Small modifications to the isoprenoid structure, such as the inclusion of bioorthogonal groups (e.g., azides, alkynes, etc.), do not perturb enzyme activity and allow for conjugation via click chemistry to different functionalities depending on the application. Similarly, *S*-prenylation mediates indispensable biological pathways and it is therefore involved in numerous diseases, including viral, bacterial, and protozoal infections.^103^

Hence, Kim et al. employed *S*-prenylation in tandem with the click chemistry methodology in the synthesis of homogeneous protein–drug conjugates.^104^ The procedure followed site-specific linkage of a toxic monomethyl auristatin F (MMAF) (a synthetic antineoplastic agent currently in use within the treatment of multiple myeloma) to repebodies (repeat antibodies, i.e., protein scaffolds with high affinity for epidermal growth factor receptor EGFR),^105^ engineered to contain a C-terminal CVIM motif (Cys-Val-Ile-Met, acting as a FTase recognition sequence). This was utilized for *S*-prenylation, followed by click-like oxime ligation with an aminooxylated MMAF (β-glucoronide linked), thereby yielding a homogeneous repebody-MMAF conjugate (or repebody derivative-drug conjugate, RDC; Figure 8A). This showed advantages in drug delivery due to the stable oxime linkage as well as high efficacy toward EFGR-positive cell lines and receptor-specific cytotoxicity; further, RDCs demonstrated *in vivo* antitumor activity in mice, though this was limited due to the short half-life of the small proteins. Further antibody–drug conjugate (ADC) synthesis and stability assessment was undertaken by Shin et al.,^106^ utilizing the same approach outlined by Kim et al., and a HER2-targeting antibody (overexpression of human epidermal growth factor receptor 2, or HER2, is a marker of breast cancer). Analysis of the drug content remaining in rat plasma accomplished by LC-MS/MS showed a staggering 85%, and *in vivo* rat studies showed the half-life of the ADC was similar to that of the market drug trastuzumab (Herceptin).

Further, Distefano et al. employed *S*-prenylation with a small protein scaffold (designed ankyrin repeat proteins, DARPins), which was engineered to bind a breast cancer cell marker (epithelial cell adhesion molecules, EpCAM) and also contained a CVIA motif (Cys-Val-Ile-Ala, acting as a FTase inhibitor).^107^ The isoprenoid analogue was chosen to contain an azide moiety, which subsequently underwent SPAAC with a dibenzocyclooctyne-functionalized fluorophore (TAMRA-DBCO). The binding affinity of the resulting conjugate to EpCAM was examined via flow cytometry, and it was found to be high toward EpCAM-overexpressing MCF-7 cells, which is a promising result for future use of this methodology in cancer research (Figure 8B).

## Conclusion and Perspective

In conclusion, click chemistry and bioorthogonal chemistry have successfully become well-established methods due to their extreme chemoselectivity and high reliability, highlighting the elegance and accessibility of this Nobel Prize-winning approach. The breadth of applications and widespread use in fields such as biotechnology and healthcare indicate a promising outlook for the future of the pharmaceutical industry and associated research.

Specific to the drug discovery and development avenue, as well as several branches of biochemistry, click chemistry has enabled significant advancements in the study and exploitation of several areas of interest, among which are the three explored in this Review (protein–drug conjugates, antisense oligonucleotides, and DNA-encoded libraries). The development of DEL technology within the context of lead compound identification showcases it as a powerful tool, particularly for investigating challenging targets with exceptional results, successfully returning quality druglike compounds as a result of screening. Furthermore, employing click chemistry with oligonucleotides has become a routine protocol. As a result, the incorporation of the triazole linkage within the DNA backbone was shown as a successful strategy within the context of DNA replication, leading to the assembly of functional genes. Click ligation of antisense oligonucleotides, with increased stability toward degradation and improved cellular uptake as a result of triazole incorporation, was used to selectively modify bases and nucleoside derivatives (LNA and G-clamp) with subsequent enhanced target binding and mismatch sensitivity. Further, the use of click chemistry alongside protein lipidation was shown to be a powerful method for site-selective protein modification, thereby allowing the construction of drug conjugates, which hold exceptional promise in cancer research.

Future developments of click methodology should aim to overcome existing limitations in its widespread clinical use, such as often mismatched physiochemical properties of compounds employed *in vivo*, which directly impact safety, pharmacokinetics, and pharmacodynamics.^108^ Given the rapid advancement of this emerging field, careful studies are expected to improve practical bioconjugation protocols to circumvent this issue. An example of this kind of endeavor is click-initiated bond-breaking reactions for drug release and delivery, and thus prodrug activation, e.g., the reaction of an arylazido group with acrolein (overproduced by tumors), generating an endogenous trigger in cancer cells.^109^

### Future Developments: Anticancer therapies

Click-to-release is an ever-expanding space in the field in prodrug development, as it allows for an exogenous trigger (enzymes such as esterases, small molecules such as thiols, and nonchemical triggers such as UV irradiation)^110^ to control the release of the active substance, minimizing adverse side effects. For example, the delivery of the cytotoxic drug doxorubicin was achieved by Brönstrup et al. utilizing the trimethyl lock (TML) lactonization in conjunction with IEDDA of a vinyl ether and a tetrazine.^111^ Doxorubicin was conjugated via amide coupling onto the vinyl-TML, which was then deprotected due to the addition–elimination of IEDDA to reveal the free phenol and liberate the autoimmolative TML core. This rapidly undergoes lactonization to the hydrocoumarin, cleaving the amide bond and releasing the drug.

Exciting perspectives include the use of click chemistry in personalized medicine, anticipating the needs of patients suffering from currently terminal illnesses. Specifically, novel modifications to the previously described site-specific protein conjugation approach pioneered by Bertozzi et al.^44^ show concrete promise in radiolabeling studies. Thus, a radioimmunoconjugate with excellent *in vivo* behavior ([^89^Zr]Zr-DFO-pertuzumab) was recently advanced to first-in-human clinical trials in patients with HER2-positive metastatic breast, gastric and bladder cancer.^112^

Furthermore, the direction of future progress of anticancer therapies seems to reach beyond monospecific antibodies, with five examples of bispecific antibodies approved by the US Food and Drug Administration and European Medicines Agency as recently as 2021.^113−115^ In order to access further advanced mechanisms of action, the generation of multiprotein drug conjugates (with small-molecule attachments) is necessary. For this reason, Bertozzi et al.^116^ has developed a new method to synthesize functionalized three-protein constructs, based on checkpoint inhibitory T cell engagers (CiTEs)^117^ with the addition of immunomodulating proteins for enhanced therapeutic benefit; notable among these are the sialidase enzyme, for removal of immunosuppressive sialic acid glycans from target and effector cells, and an anti-PD-1 Fab checkpoint inhibitor (where Fab describes antigen binding fragments of PD-1, or programmed cell death 1, denoting a coinhibitory receptor expressed on the surface of T-cells to negatively regulate immune response).^118^ These constructs were achieved via tetrazine-bicyclononyne strain-promoted IEDDA, with each CiTE containing a small molecule also conjugated via SPAAC for imaging.^116^ The future of cancer therapies seems to heavily rely on bioorthogonal chemistry, which showcases the far-reaching impact of this promising novel technology.
